# Dynamic development of metabolic syndrome and its risk prediction in Chinese population: a longitudinal study using Markov model

**DOI:** 10.1186/s13098-018-0328-3

**Published:** 2018-04-02

**Authors:** Xiaoxian Jia, Qicai Chen, Peipei Wu, Meng Liu, Xiaoxiao Chen, Juan Xiao, Lili Chen, Pengpeng Zhang, Shumei Wang

**Affiliations:** 1 Department of Epidemiology, School of Public Health, Shandong University, 44 Wenhua West Road, Jinan, 250012 China; 2 Department of Prevention and Health Care, Dongying Shengli Oilfield Central Hospital, Dongying, 257000 China; 3 Department of Commodity Price and Medical Insurance, Shenzhen People’s Hospital, Shenzhen, 518020 China; 4 Department of Hospital Infection Management, Qilu Hospital of Shandong University, Jinan, 250012 China; 5 Department of Medical Records and Statistics, Zhejiang Hospital, Hangzhou, 310013 China; 6 Center of Evidence-based Medicine, The Second Hospital of Shandong University, Jinan, 250033 China; 7 Department of Nutrition and Food Safety, Zhejiang Center for Disease Control and Prevention, Hangzhou, 310051 China; 8 Tianjin Entry-Exit Inspection and Quarantine Bureau, Tianjin, 300000 China

**Keywords:** Metabolic syndrome, Markov model, Longitudinal studies

## Abstract

**Background:**

With the increasing prevalence of metabolic syndrome (MS), there is a need to track and predict the development of MS. In this study, we established a Markov model to explore the natural history and predict the risk of MS.

**Methods:**

A total of 21,777 Chinese individuals who had at least two consecutive health check-ups between 2010 and 2015 were studied. MS was defined using the Chinese Diabetes Society criteria. Twelve metabolic abnormal states (the no component state, four isolated component states, six 2-component states, and the MS state) were contained in each Markov chain. The transition probability was the mean of five probabilities for the transition between any two states in 2 consecutive years.

**Results:**

The dyslipidemia or overweight/obesity components were most likely to initiate the progress of MS in individuals aged 18–49. However, for individuals over 50 years old, the most likely initiating component of MS was dyslipidemia or hypertension. People who initially had dyslipidemia were most likely to develop the combined state of dyslipidemia with overweight/obesity before the age of 50, but after 50 years of age, the state of dyslipidemia merged with hypertension was the most common. Subjects (with the exception of males over 50 years of age who initially had an isolated state of hyperglycemia) who initially had an isolated state of overweight/obesity, hypertension, or hyperglycemia were most likely to develop a combination of one of these initial states with dyslipidemia. Males who initially had isolated hyperglycemia tended to develop hypertension after age 50. There was a greater chance for subjects who initially had an isolated hyperglycemia state or 2-component state that contained hyperglycemia to develop MS within 10 years compared to those who initially had other abnormal metabolic states.

**Conclusions:**

The occurrence of MS primarily began with overweight/obesity or dyslipidemia in people aged 18–49. However, for those over 50 years old, MS primarily initiated under the conditions of dyslipidemia or hypertension. When MS started under the conditions of overweight/obesity, hypertension or hyperglycemia, dyslipidemia tended to occur next. People who initially had isolated hyperglycemia or a 2-component state that contained hyperglycemia had a higher risk of developing MS than those with other initiating states.

## Background

Metabolic syndrome (MS) is a cluster of metabolic abnormalities, including obesity, hypertension, dyslipidemia and hyperglycemia [1]. MS has become a global public health issue, and many scholars have devoted attention to it [2–7]. Previous studies focused on searching for risk factors and early biomarkers of MS [8–13]. However, as a dynamic developing process, the natural history of MS has not yet been determined. Although some researchers have explored the occurrence and longitudinal changes of MS over time, controversy still exists regarding this question [14–16].

In 2004, the Chinese Diabetes Society (CDS) proposed diagnostic criteria for MS for Chinese adults [17]. According to the CDS criteria, MS included four components: overweight or obesity, hypertension, dyslipidemia, and hyperglycemia; patients who had at least three simultaneous components were diagnosed as having MS. Depending on how many components the individual has, subjects can be divided into twelve different states. That is, one state without components, four states of isolated components, six states of a 2-component combination, and one state of MS. The development of MS is very complex. Each individual over a certain period can transfer to other states or maintain their original state; moreover, there are a total of 144 types of transition. It remains unclear which component triggers the cascade of metabolic disorders, which components will appear next, whether each component occurs simultaneously or successively and to what extent each component plays a role in the development of MS. Therefore, it is necessary to explore the natural history of MS and identify the components that initiate and promote the development of MS. For healthy people, the initiation of MS should be prevented, while for patients with metabolic disorders, prevention and control of the component that accelerates the development of MS could potentially arrest the continuing development of MS.

As a well-recognized method of simulating the natural history of chronic diseases, Markov models have been used in previous studies to explore the development of MS. Hwang et al. applied a Markov model to describe and predict the development of MS that focused on young people aged 18–45 years in Taiwan. Their findings confirmed that the development of MS was different in men and women [18]. In 2014, our research group included a total of 7510 subjects and constructed a 7-state Markov model to investigate the natural development of MS in different gender and age groups, with the results indicating that there were gender and age differences in the progression of MS [19]. However, the limitation of this study was that the six states were composed of a 2-component condition were not completely separated. The states composed of 2-component conditions are an important part of the natural history of MS. Without further study of the dynamic changes of the 2-component states, the entire pathogenic process of MS remains unknown. Accordingly, this study established a 12-state Markov model based on the CDS criteria in different gender and age groups, with 21,777 subjects selected from a 6-year history of health check-ups, to delineate the natural history and calculate a risk prediction of MS, which is of great significance for preventing and controlling MS.

## Methods

### Study participants

Subjects who had undergone routine health check-ups at the Health Management Center of a general hospital in Dongying City were recruited from December 2010 to December 2015. Subjects who were aged 18–88 years, without restrictions on gender or occupation, and had at least 2 consecutive years of routine health check-ups were considered (n = 22,584). Those who had coronary heart diseases (CHD), type I diabetes, familial hyperlipidemia, or lacked the necessary information were excluded from this study (n = 807). Finally, a total of 21,777 subjects were included in our study. Because of the different cohort entrance timeframes, the first health check-up could occur in any year in the interval of 2010–2014; therefore, data from the first year for each subject was used as the baseline data. In the preliminary analysis, individuals were divided into four age groups, the 18–39, 40–49, 50–59 and ≥ 60 year group, and were further divided into two genders. We found that people under 50 years of age and people over 50 years of age had different processes of MS and that there was a significant difference in the prevalence of MS and its components between men and women. Finally, subjects were divided into the 18–49 years of age group and ≥ 50 years of age group for each gender.

### Clinical and biochemical measurements

Every participant underwent a doctor’s interview, anthropometric measurements and blood biochemical analysis. The doctors’ interview collected general information about the subjects, including age, medical history, and history of familial hyperlipidemia. The medication use was included in the medical history referred to whether the subjects were taking anti-hypertensive drugs, anti-hyperglycemic drugs, and so on. Weight, height, and blood pressure were measured using a certified measurement instrument. Subjects’ height and weight were measured while wearing light clothing, without shoes, and with an empty bladder. BMI was calculated as weight (kg) divided by height (m) squared. Blood pressure was measured twice using a calibrated mercury sphygmomanometer on the right arm after subjects were seated comfortably in a chair for at least 5 min. After fasting for 12 h, blood samples were collected from the upper arm vein to test fasting plasma glucose (FPG), triglyceride (TG) and high-density lipoprotein cholesterol (HDL-C). FPG was measured by the glucose oxidase method, and TG and HDL-C were measured by an enzymatic chemical test.

### Definition of metabolic syndrome

In this study, the CDS criteria was adopted to define MS according to the following categories: overweight or obesity (BMI ≥ 25.0 kg/m^2^); hypertension [systolic blood pressure (SBP) ≥ 140 mmHg or diastolic blood pressure (DBP) ≥ 90 mmHg] or the use of anti-hypertensive drugs for previously diagnosed hypertension; and dyslipidemia [fasting TG ≥ 1.7 mmol/L (110 mg/dL) or fasting HDL-C (< 0.9 mmol/L in men, < 1.0 mmol/L in women)]; hyperglycemia [FPG ≥ 6.1 mmol/L (110 mg/dL) or 2 h post-meal glucose (PG) ≥ 7.8 mmol/L (140 mg/dL)] or the use of anti-hyperglycemic drugs for previously diagnosed hyperglycemia. The diagnosis of each component was based on the above criteria in this study. Subjects with at least three of the four components were diagnosed with MS.

### Statistical analysis

#### Establishment of the Markov model

**Fig. 1:**
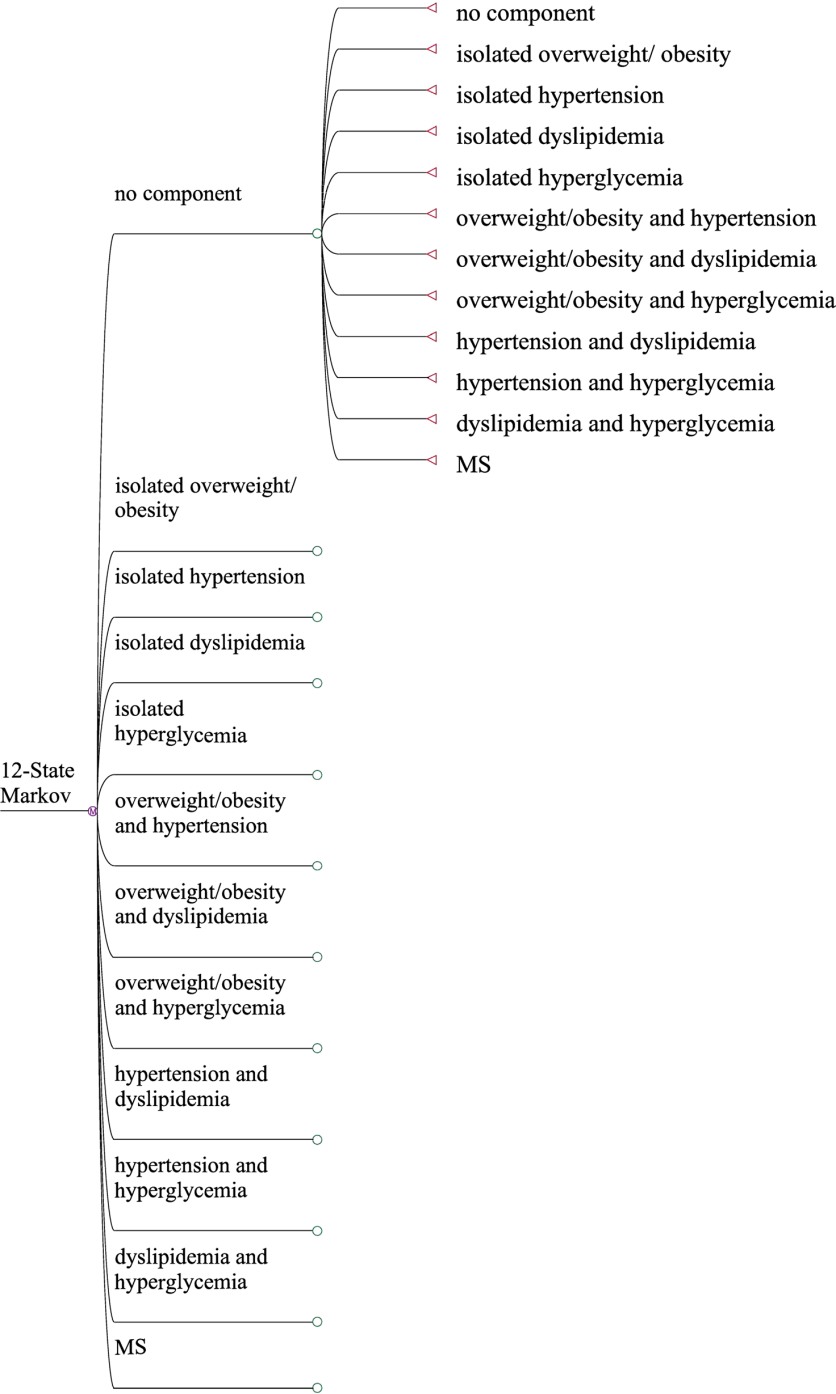
A 12-state Markov model used to describe the progression of metabolic components. Subjects may be in the no component state, and 1 year later, they can transit into the other eleven states, or remain in the no component state

In our study, a 12-state Markov model was established based on the developing process of MS. Each Markov chain included no component state, isolated overweight/obesity state, isolated hypertension state, isolated dyslipidemia state, isolated hyperglycemia state, overweight/obesity and hypertension state, overweight/obesity and dyslipidemia state, overweight/obesity and hyperglycemia state, hypertension and dyslipidemia state, hypertension and hyperglycemia state, dyslipidemia and hyperglycemia state, and MS state. The graphical presentation of the 12-state Markov model is shown in Fig. 1. In the cycle-based Markov model, the time processing unit was 1 year. Before the start of the cycle, each subject could only be in any of the 12 states but could not be in two states at the same time, that is, states were mutually exclusive and collectively exhaustive. After 1 year of the cycle, subjects could be transferred from one state to another or remain in their original state. A reversible multistate Markov model was designed to study all of the possible transitions from one state to another in MS. As shown in Fig. 1, subjects can be in the no component state, and 1 year later, they can transit into the other eleven states, or remain in the no component state. Subjects can also be in the isolated overweight/obesity state or the MS state, in which they can remain or transit into another state after 1 year.

#### Calculation of the transition probabilities and predictive rates of MS

The transition probability was used to describe the possibility of transitioning between states since the state transition was random in the Markov model. In this study, the annualized probability of transitioning was calculated in each age and gender group to indicate the transition probability from one state to another or to maintain the initial state in every Markov cycle. Five Markov cycles were contained in our 6-year health check-up data. To maximize the reliability of the results, the mean value of the five annualized probabilities of transitioning was used as the transition probability in the Markov model. The results were then incorporated into the 12-state Markov model to predict the effect of different initial states on the development of MS after 10 years in each gender and age group. To simplify the research, a series of assumptions were necessary for the Markov model: (1) For a Markov process, the future state depended only on the subject’s current state without any association with the prior state (Markov chain assumption). (2) Each baseline transition probability in each Markov cycle was considered to be constant during the follow-up period.

#### Additional statistical methods

The baseline characteristics were described statistically. The mean ± standard deviations and proportions were used to express the normally distributed continuous data and categorical data, respectively. The Chi square test was used to compare differences for the categorical data. A two-sided *p* value of less than 0.05 was considered as statistically significant in the analysis.

All of the above statistical analyses and calculations of the transition probabilities were performed using SPSS software (version 24.0, SPSS, Chicago, IL, USA). The risk prediction of MS in the Markov model was performed using TreeAge Pro 2011 (Tree Age Software, Inc., Williamstown, MA). The Figures of the predictive rates were generated using R software (version x64 3.3.3, R Foundation for Statistical Computing, Vienna, Austria).

## Results

### Participant characteristics at baseline and follow-up visits

The baseline characteristics of the subjects stratified by gender and age are shown in Table 1. Among the 21,777 subjects included in this study, there were 14,250 men (65.44%) and 7527 women (34.56%) aged 18–88 years old. After subgrouping by age, the 18–39 years age group included 4730 men and 3271 women, the 40–49 years age group included 5088 men and 2705 women, the 50–59 years age group included 2678 men and 892 women, and the ≥ 60 years age group included 1754 men and 659 women. The prevalence of hypertension, hyperglycemia and MS increased with age in both men and women. The prevalence of overweight/obesity or dyslipidemia had the same trend in women but not in men.

**Table 1:** Basic characteristics of the study sample stratified by gender and age

Basic survey	18–39 (years)	40–49 (years)	50–59 (years)	≥ 60 (years)	*x* ^2^	p*
Men						
N	4730	5088	2678	1754		
Age (years)	32.11 ± 4.99	44.45 ± 2.92	54.23 ± 2.95	68.39 ± 6.42		
Overweight or obesity	2220 (46.93%)	2781 (54.66%)	1513 (56.50%)	933 (53.19%)	85.382	< 0.0001
Hypertension	981 (20.74%)	1821 (35.79%)	1241 (46.24%)	1069 (60.95%)	1096.064	< 0.0001
Dyslipidemia	1790 (37.84%)	2554 (50.20%)	1300 (48.54%)	772 (44.01%)	167.648	< 0.0001
Hyperglycemia	193 (4.08%)	627 (12.32%)	582 (21.73%)	501 (28.56%)	869.355	< 0.0001
MS	489 (10.34%)	1080 (21.23%)	731 (27.30%)	568 (32.38%)	535.283	< 0.0001
Women						
N	3271	2705	892	659		
Age (years)	31.45 ± 5.14	44.32 ± 2.88	53.46 ± 2.85	66.92 ± 5.22		
Overweight or obesity	418 (12.78%)	484 (17.89%)	295 (33.07%)	320 (48.56%)	640.497	< 0.0001
Hypertension	193 (5.90%)	338 (12.50%)	241 (27.02%)	383 (58.12%)	1263.240	< 0.0001
Dyslipidemia	509 (15.56%)	567 (20.96%)	357 (40.02%)	319 (48.41%)	490.266	< 0.0001
Hyperglycemia	72 (2.20%)	92 (3.40%)	81 (9.08%)	149 (22.61%)	506.689	< 0.0001
MS	63 (1.93%)	82 (3.03%)	96 (10.76%)	180 (27.31%)	750.878	< 0.0001

The mean ± standard deviations and percentages were used to express the normally distributed continuous variables and the categorical variables, respectively. Chi square test was used to compare the differences for categorical variables, the test statistics are *x*^*2*^ values

* *p* for each row testing the null hypothesis that values for four age groups were equal. Two-sided p < 0.05 was considered statistically significant

The general information of the study cohort is shown in Table 2. The total follow-up period was 65,847 person-years, and the median was 2.77 years. The prevalence of MS on the baseline is 15.10%, with 19.93, 23.45, 26.90, 29.82, 32.75% in the follow-up years. The prevalence of MS increased over time. At the same time, the prevalence of hypertension, dyslipidemia and hyperglycemia also increased annually.

**Table 2:** General information of the six-year follow-up cohort

Variables	Baseline	1-year follow-up	2-year follow-up	3-year follow-up	4-year follow-up	5-year follow-up	p*
	(N = 21,777)	(N = 19,330)	(N = 11,226)	(N = 8652)	(N = 7918)	(N = 4776)	
Age (years)	43.98 ± 12.36	44.72 ± 12.36	45.57 ± 11.62	47.28 ± 11.86	48.47 ± 11.35	49.96 ± 11.31	< 0.0001
Sex (male)	14,250 (65.44%)	12,504 (64.69%)	7522 (67.00%)	5432 (62.76%)	4918 (62.11%)	3086 (64.61%)	< 0.0001
Overweight or obesity	8964 (41.16%)	8312 (43.00%)	5031 (44.82%)	3732 (43.13%)	3542 (44.73%)	2174 (45.52%)	< 0.0001
Hypertension	6267 (28.78%)	7125 (36.86%)	4781 (42.59%)	4025 (46.52%)	3864 (48.86%)	2478 (51.88%)	< 0.0001
Dyslipidemia	8168 (37.51%)	8208 (42.46%)	4880 (43.47%)	3906 (45.15%)	4301 (54.32%)	2927 (61.29%)	< 0.0001
Hyperglycemia	2297 (10.55%)	2530 (13.09%)	1780 (15.86%)	1516 (17.52%)	1555 (19.64%)	1019 (21.34%)	< 0.0001
MS	3289 (15.10%)	3852 (19.93%)	2632 (23.45%)	2328 (26.90%)	2361 (29.82%)	1564 (32.75%)	< 0.0001

The mean ± standard deviations and percentages were used to express the normally distributed continuous variables and the categorical variables, respectively

* *p* for each row testing the null hypothesis that values for 6 years were equal

### Transition probabilities stratified by gender and age

The transition probabilities are presented in Tables 3, 4, 5, 6. The transition probabilities from the no component state or any state with isolated components to the MS state were higher in men than in women, and the probability of maintaining the MS state was higher in men in both age groups. However, as age increased, the gap gradually narrowed. Among the eleven other states that the no component state can transition to, the state of isolated dyslipidemia and isolated overweight/obesity had the highest probability of being transition destinations in the group of males and females aged between 18 and 49. However, in subjects over 50 years old, the highest transition probabilities occurred in the isolated dyslipidemia state and isolated hypertension state, and the above probabilities of transitioning in the two age groups were higher in men than in women.

**Table 3:** Transition probabilities (%) in Markov models for men in the 18–49 year group

Starting state	State after transition											
	No component	Isolated overweight or obesity	Isolated hypertension	Isolated dyslipidemia	Isolated hyperglycemia	Overweight or obesity and hypertension	Overweight or obesity and dyslipidemia	Overweight or obesity and hyperglycemia	Hypertension and dyslipidemia	Hypertension and hyperglycemia	Dyslipidemia and hyperglycemia	MS
No component	66.44	10.15	4.19	10.78	0.67	1.47	3.28	0.13	1.13	0.12	0.36	1.28
Isolated overweight or obesity	7.14	47.19	1.12	2.70	0.16	8.78	22.66	1.23	0.32	0.03	0.03	8.63
Isolated hypertension	0	0	63.81	0	0	9.61	0	0	16.29	2.08	0	8.21
Isolated dyslipidemia	23.04	3.14	2.85	48.02	0	1.18	9.47	0	4.96	0	1.71	5.61
Isolated hyperglycemia	0	0	0	0	45.66	0	0.	6.24	0	7.99	28.34	11.76
Overweight or obesity and hypertension	0	0	7.24	0	0	66.12	0	0	2.43	0.30	0	23.91
Overweight or obesity and dyslipidemia	3.14	15.08	0	4.86	0	2.44	58.53	0.64	0.41	0	0.06	14.83
Overweight or obesity and hyperglycemia	0	0	0	0	7.74	0	0	60.11	0	1.98	2.97	27.20
Hypertension and dyslipidemia	0	0	26.15	0	0	5.68	0	0	53.36	0.99	0	13.82
Hypertension and hyperglycemia	0	0	0	0	0	0	0	0	0	79.20	0	20.80
Dyslipidemia and hyperglycemia	0	0	0	0	24.62	0	0	0.42	0	4.87	56.02	14.07
MS	0	0	1.91	0	0.23	11.36	0	4.04	3.40	3.19	0.64	75.25

**Table 4:** Transition probabilities (%) in Markov models for women in the 18–49 year group

Starting state	State after transition											
	No component	Isolated overweight or obesity	Isolated hypertension	Isolated dyslipidemia	Isolated hyperglycemia	Overweight or obesity and hypertension	Overweight or obesity and dyslipidemia	Overweight or obesity and hyperglycemia	Hypertension and dyslipidemia	Hypertension and hyperglycemia	Dyslipidemia and hyperglycemia	MS
No component	78.21	7.15	2.44	8.45	0.60	0.46	1.30	0.01	0.49	0.06	0.20	0.63
Isolated overweight or obesity	18.51	52.01	0.95	4.32	0.63	4.98	15.19	0.21	0	0	0.12	3.07
Isolated hypertension	0	0	74.82	0	0	4.98	0	0	15.39	1.45	0	3.37
Isolated dyslipidemia	39.90	1.37	2.04	46.36	0	0.55	4.62	0	2.87	0	0.56	1.72
Isolated hyperglycemia	0	0	0	0	67.46	0	0	0.54	0	13.09	14.69	4.22
Overweight or obesity and hypertension	0	0	17.75	0	0	58.62	0	0	4.67	2.64	0	16.32
Overweight or obesity and dyslipidemia	9.33	21.16	0	12.99	0.34	4.12	42.34	0	0.62	0	1.51	7.57
Overweight or obesity and hyperglycemia	0	0	0	0	17.37	0	0	58.88	0	0.97	1.36	21.42
Hypertension and dyslipidemia	0	0	23.20	0	0	2.98	0	0	62.53	0.43	0	10.87
Hypertension and hyperglycemia	0	0	0	0	0	0	0	0	0	79.44	0	20.56
Dyslipidemia and hyperglycemia	0	0	0	0	25.91	0	0	5.26	0	3.90	48.53	16.40
MS	0	0	13.52	0	3.90	11.34	0	2.36	3.36	3.64	1.23	60.64

**Table 5:** Transition probabilities (%) in Markov models for men in the ≥ 50 year group

Starting state	State after transition											
	No component	Isolated overweight or obesity	Isolated hypertension	Isolated dyslipidemia	Isolated hyperglycemia	Overweight or obesity and hypertension	Overweight or obesity and dyslipidemia	Overweight or obesity and hyperglycemia	Hypertension and dyslipidemia	Hypertension and hyperglycemia	Dyslipidemia and hyperglycemia	MS
No component	60.42	5.09	12.11	11.44	2.26	1.29	2.00	0.62	1.68	0.74	0.81	1.54
Isolated overweight or obesity	8.97	40.96	1.65	1.62	0	14.66	18.76	3.47	0.21	0	0	9.70
Isolated hypertension	0	0	62.64	0	0	7.29	0	0	16.48	6.03	0	7.56
Isolated dyslipidemia	23.89	2.32	3.17	41.93	0	0.90	5.23	0	11.27	0	2.66	8.63
Isolated hyperglycemia	0	0	0	0	47.32	0	0	4.79	0	20.63	12.86	14.41
Overweight or obesity and hypertension	0	0	9.28	0	0	60.14	0	0	1.86	1.24	0	27.49
Overweight or obesity and dyslipidemia	3.10	13.77	0	3.78	0.25	3.74	52.87	1.47	1.87	0	0.49	18.65
Overweight or obesity and hyperglycemia	0	0	0	0	7.03	0	0	56.40	0	2.34	2.73	31.49
Hypertension and dyslipidemia	0	0	21.42	0	0	2.76	0	0	57.00	2.60	0	16.22
Hypertension and hyperglycemia	0	0	0	0	0	0	0	0	0	73.47	0	26.53
Dyslipidemia and hyperglycemia	0	0	0	0	20.75	0	0	1.16	0	1.39	52.44	24.26
MS	0	0	0.83	0	0.10	10.74	0	3.07	2.40	1.82	0.60	80.43

**Table 6:** Transition probabilities (%) in Markov models for women in the ≥ 50 year group

Starting state	State after transition											
	No component	Isolated overweight or obesity	Isolated hypertension	Isolated dyslipidemia	Isolated hyperglycemia	Overweight or obesity and hypertension	Overweight or obesity and dyslipidemia	Overweight or obesity and hyperglycemia	Hypertension and dyslipidemia	Hypertension and hyperglycemia	Dyslipidemia and hyperglycemia	MS
No component	64.86	4.01	8.17	11.30	2.04	0.51	2.19	0.65	2.69	0.40	1.67	1.50
Isolated overweight or obesity	12.08	40.30	0.78	5.38	2.46	11.50	15.70	4.24	0	0	0.62	6.93
Isolated hypertension	0	0	56.31	0	0	8.60	0	0	23.91	5.02	0	6.17
Isolated dyslipidemia	23.89	1.96	1.92	52.69	2.22	1.33	3.11	0	5.32	0	1.96	5.61
Isolated hyperglycemia	0	0	0	0	55.98	0	0	0	0	13.05	19.09	11.87
Overweight or obesity and hypertension	0	0	6.47	0	0	67.58	0	0	4.83	0.54	0	20.58
Overweight or obesity and dyslipidemia	6.22	13.99	0	6.42	0	5.09	48.73	0	2.29	0	0	17.27
Overweight or obesity and hyperglycemia	0	0	0	0	10.61	0	0	50.35	0	4.74	7.12	27.18
Hypertension and dyslipidemia	0	0	20.05	0	0	0.32	0	0	61.15	3.42	0	15.07
Hypertension and hyperglycemia	0	0	0	0	0	0	0	0	0	75.01	0	24.99
Dyslipidemia and hyperglycemia	0	0	0	0	9.62	0	0	0	0	2.09	63.02	25.28
MS	0	0	6.04	0	2.14	9.63	0	0.58	4.80	0.95	0.35	75.52

In the 18–49 years age group, subjects who were initially in the isolated dyslipidemia state had a higher transition probability to develop a combination of this state with overweight/obesity state, whereas for those over 50 years old, the transition probability was higher in the combination of this state with the hypertension state. Subjects (with the exception of males above 50 years of age who initially had an isolated state of hyperglycemia) who initially had an isolated state of overweight/obesity, hypertension or hyperglycemia had a higher transition probability to develop the next component, dyslipidemia. A higher transition probability from an isolated hyperglycemia state to the state of hypertension and hyperglycemia was observed in men over 50 years old.

### Risk prediction of the development of MS initiated from the states without components, any isolated component, or any 2-component combination

**Fig. 2:**
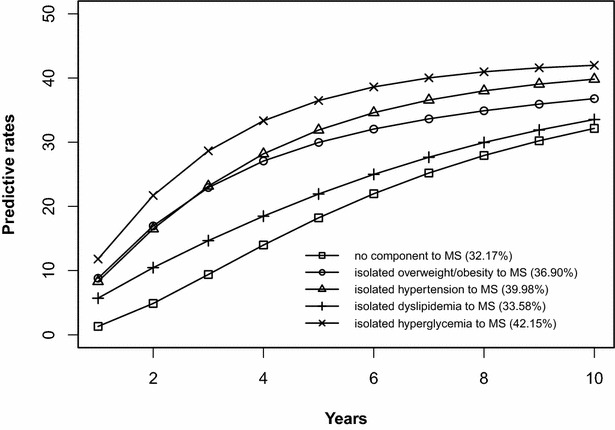
Risk prediction of MS according to various starting components in men aged 18–49

**Fig. 3:**
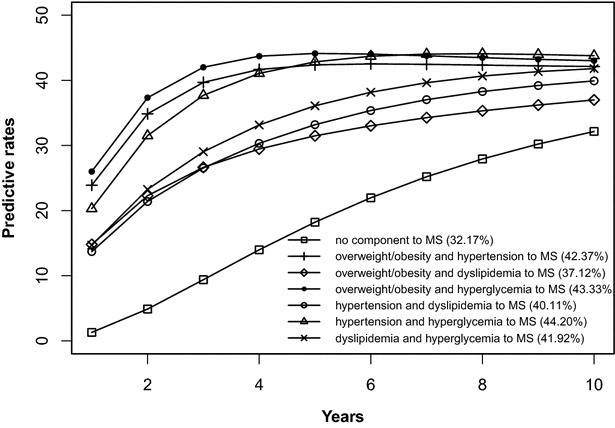
Risk prediction of MS according to various starting components in men aged 18–49

**Fig. 4:**
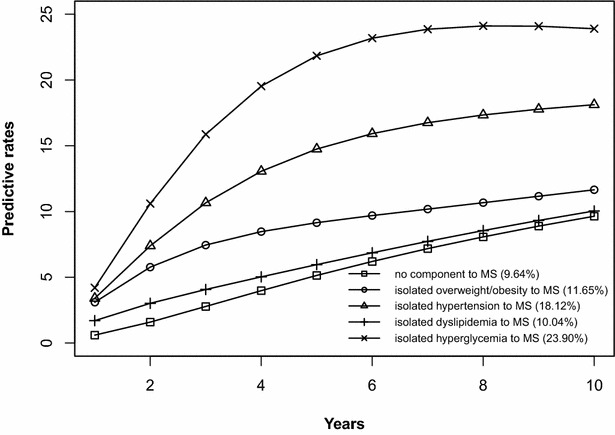
Risk prediction of MS according to various starting components in women aged 18–49

**Fig. 5:**
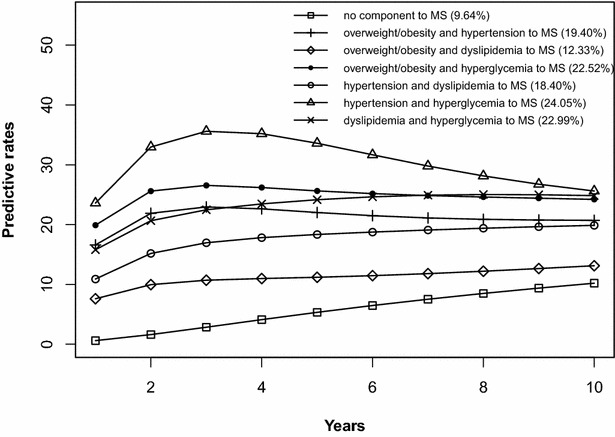
Risk prediction of MS according to various starting components in women aged 18–49

**Fig. 6:**
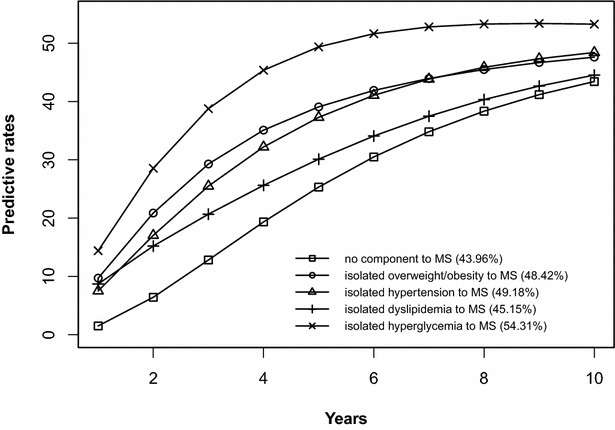
Risk prediction of MS according to various starting components in men in after age 50

**Fig. 7:**
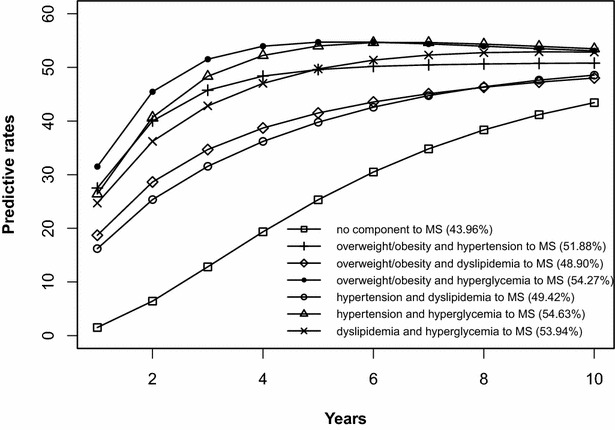
Risk prediction of MS according to various starting components in men in after age 50

**Fig. 8:**
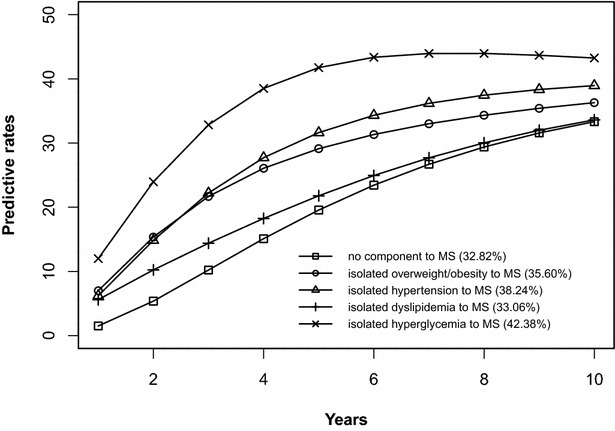
Risk prediction of MS according to various starting components in women after age 50

**Fig. 9:**
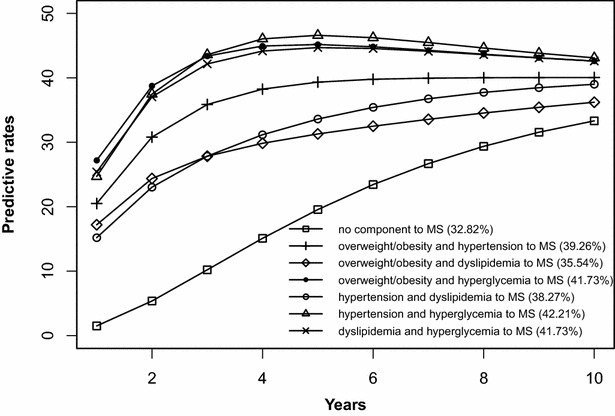
Risk prediction of MS according to various starting components in women after age 50

The risk prediction of the development of MS after 10 years is shown in Figs. 2, 3, 4, 5, 6, 7, 8, 9. The abscissa represents the time after the initiation of different states; the ordinate represents the predictive rate of MS. Icons are used to represent different starting states, and each icon on the curve represents the predictive rate of each year. The predictive rates in the first year are the transition probabilities calculated in the previous step. The predictive rate in the future is calculated by the Markov model based on the transition probabilities. Figures 2, 4, 6, and 8 are the predictive rates of starting in the no component state or any state with isolated components. Figures 3, 5, 7, and 9 are the predictive rates of starting in the no component state or any 2-component state divided by gender and age.

In two age groups, males had a higher transition probability from the no component state to the MS state compared to females, but the gap was significantly smaller after the age of 50 years old. There was a greater possibility for subjects who began in the isolated hyperglycemia state to develop MS within 10 years than those who started in other isolated component states. Similarly, subjects beginning in a 2-component state that included hyperglycemia were more likely to develop MS after 10 years than those starting in a 2-component state that did not include hyperglycemia.

### Validation of the Markov model

The estimated prevalence of MS after 5 years in men and women aged 18–49 years who were initially in the no component state was 18.22 and 5.33%, respectively. The prevalence from empiric data was 21.90% in men and 6.15% in women. In addition, we compared other estimated prevalences with empiric data, and the results were similar, indicating that the Markov model used in this study was effective in predicting disease.

## Discussion

In this study, a Markov model was applied to investigate the development of MS in a large-scale Chinese population. The advantage of this model is that a disease can be divided into several states and that these states can be transferred from one to another so that the development of a disease can be simulated [20, 21]. The Markov model has been used to study the history of chronic diseases in previous studies. For example, by constructing a Markov model, Kabat et al. followed subjects with different obesity phenotypes over time to examine the changes in their metabolic profiles [22]. Chao et al. simulated the natural history of diabetes and assessed the long-term effects of a health management program on diabetic patients [23]. In our research, a 12-state Markov model was constructed in each gender and age group to describe and predict the natural occurrence and development process of MS.

The development of MS is likely a process in which each component appears in succession. We believe that the first component that occurred with the highest incidence is most likely to initiate MS, and it is a beneficial insight to identify the initiating component of MS. To date, controversy still exists regarding this question [18, 24, 25]. It may be a result of the different gender and age groups adopted in studies. The results of this study showed that MS may be initiated in dyslipidemia or overweight/obesity in the respective groups of men and women aged 18–49. However, in addition to dyslipidemia, another possible initiating component of MS was the change from overweight/obesity to hypertension in both men and women over 50 years old. According to these results, we concluded that dyslipidemia was one of the first components to appear among the population. At present, improvements in living standards and changes in dietary habits coupled with an excessive energy intake and less exercise greatly increase the incidence of dyslipidemia. In addition, dyslipidemia can mediate free fatty acids (FFA) to damage the islet function, which may lead to hypertension and hyperglycemia [26, 27]. Overweight/obesity may be another initiating component of MS among people under the age of 50 in addition to dyslipidemia. Evidence that obesity is an important risk factor for hypertension, dyslipidemia and hyperglycemia has been reported [28]. Some studies also indicated that obesity, especially abdominal obesity, was the most prevalent cause of MS [29]. There are several reasons that explain how the process of MS most likely begins with hypertension instead of overweight/obesity for those over the age of 50 years old. With increasing age, especially after menopause, recession of the endocrine regulation system means that the hormone levels have changed [30]. Research has shown that the development of hypertension is closely related to the endogenous sex hormones in postmenopausal women [31]. Moreover, aging exerts a marked effect on large arteries, and the degree of arterial stiffness is closely related to the occurrence of hypertension [32]. Studies that attempt to identify the initiating components of MS are very limited and have not yet reached a unanimous conclusion; therefore, the issue warrants continued investigation.

The process of the development from an isolated component state to a 2-component state was explored in this study. This study will provide important insight into identifying the pathway to MS. The results showed that people who initially had dyslipidemia were more likely to develop this state combined with the state of overweight/obesity before the age of 50. A recent study has suggested the positive association between obesity and the high prevalence of dyslipidemia [33]. We found that dyslipidemia appeared first, followed by overweight/obesity, which may occur because there are coexisting risk factors causing the simultaneous occurrence of the two components, but only dyslipidemia occurs first. It may also be that dyslipidemia can lead to overweight/obesity. The causality between the two components remains to further study. After 50 years of age, people who initially had dyslipidemia were more likely to have this condition merged with hypertension. A prospective study has shown that dyslipidemia may lead to the occurrence of hypertension in men [34]. Although the specific biological mechanisms of the above two associations have not yet been established, we propose that controlling blood lipid levels would be helpful in preventing overweight/obesity and hypertension to a certain extent. Our research also has shown that subjects (with the exception of males above 50 years of age who initially had an isolated state of hyperglycemia) who initially had an isolated state of overweight/obesity, hypertension, or hyperglycemia were most likely to develop a combination of one of these isolated states with dyslipidemia. Many studies have shown that weight gain, elevated blood pressure and blood glucose were important risk factors for dyslipidemia [35, 36]. The above results showed that dyslipidemia may play a significant role in the initiation and development of MS. However, after developing hyperglycemia, men above 50 years of age were more prone to exhibit the next component, hypertension. Other related work has shown that more than two-thirds of people with type 2 diabetes suffer from hypertension, and the development of hypertension coincides with hyperglycemia [37]. Therefore, it is essential to prevent MS in different gender and age groups according to their pathogenetic characteristics.

In this study, risk prediction of MS was calculated for individuals who initiated in any isolated component state or in a 2-component state in each gender and age group; we believe that the component that results in the highest incidence of MS may have a pivotal role in the progression of MS. In the predictions, subjects who were initially in the isolated hyperglycemia state had the highest transition probability of MS compared to the other isolated component states. At the same time, subjects who began in the 2-component state that included hyperglycemia had a greater probability to develop MS than those without hyperglycemia. These results may demonstrate that the elevated blood glucose levels is an important factor in promoting the development of MS. Although the pathogenesis of MS is not yet clear, the components of MS always presented together, indicating that this phenomenon of coexistence may be dominated by a common mechanism. Until now, most researchers have believed that insulin resistance (IR) is the central cause of MS etiology [38]. IR and islet β-cell dysfunction are the main pathophysiological mechanisms of hyperglycemia. Clinically, hyperglycemia often occurs with obesity, dyslipidemia and hypertension simultaneously or successively. Hyperglycemia may mediate the synthesis and decomposition of FFA through insulin resistance, leading to dyslipidemia; therefore, the underlying cellular and molecular mechanisms require further study [39]. In addition, elevated blood glucose levels may increase blood pressure by interfering with the reabsorption of renal tubules and excitability of the sympathetic nervous system [40, 41]. However, whether hyperglycemia can directly lead to obesity remains to be studied. In the process of prevention and control of MS, we need to pay special attention to changes in blood glucose levels. On the one hand, we should actively perform the three levels of diabetes prevention, including maintaining a healthy lifestyle and strengthening physical exercise. On the other hand, when blood glucose levels are elevated, we should take active measures to reduce blood glucose and prevent the emergence of other MS components.

In addition to the above findings, other information from the prediction probabilities was equally important. People in the no component state were also more likely to develop MS after 10 years, especially women older than 50 years of age, for whom the risk of MS tripled compared to that of young women. Chedraui et al. found that the serum marker levels of inflammation and endothelial dysfunction were mainly related to the high incidence of MS in postmenopausal women [42]. When MS started from any state with isolated components, the probabilities of developing MS after 10 years were higher than those of the no component state. The prediction probabilities may reflect the effect of each component on the development of MS. Although the predictive rate of MS initiated from the state of isolated dyslipidemia after 10 years was only one percent higher than that initiated from the state without components, dyslipidemia was a possible initiation component of MS and tended to occur simultaneously with the other components. Other work has indicated that intensified efforts at screening and treatment for dyslipidemia are warranted [43, 44]. Moreover, during the first 5 years after beginning in any 2-component state, the predictive probability of MS increased rapidly, and some probabilities seem to have peaked. Because each component of MS occurs successively or simultaneously in a short period of time, not only will the risk of cardiovascular disease and all-cause mortality increase but also the social and economic burden will increase [45, 46].

Our research tracked the progress of MS and compared the effect of different initial states on the future development of MS. According to the above characteristics, the progress of MS can be divided into four stages, namely, the health, initiation, transition and MS stages. Current guidelines and studies suggest that individuals with metabolic disorders at the premorbid state should first change their lifestyle [47–49]. This study confirms the difference in the components initiating MS between two age groups and suggests that targeted measures should be undertaken to control the corresponding components. After the occurrence of metabolic abnormalities, health promotion behaviors should also be continued and combined with drug treatment. Special attention should be paid to the control of blood glucose levels, which may affect the rapid progress of MS. Our study explored several important links in the development of MS, and we believe that this research will be of great significance for the prevention of MS in the Chinese population

## Limitations

There are still some limitations in this study. First, the 12-state Markov model assumed that the baseline transition probability of each state remained constant during the follow-up period. However, MS will be affected by many factors in the development process, such as medication or changing lifestyles. In addition, this research has not yet taken into account these factors because of the lack of a sufficient sample size and requirement of specific information. Second, the subjects in this study were recruited from the Health Management Center of the Hospital, and they were mainly in-service or retired staff; underrepresented groups, such as farmers, indicate that the study may not fully represent the general population, so extrapolation of this research conclusion requires caution. Finally, due to the limited sample size, this study focused on the changes in the states prior to the diagnosis of MS; therefore, we did not consider the four 3-component states after MS occurs. In a subsequent study, all of the states of MS can be divided to ensure that all of the natural processes of MS are studied.

## Conclusions

MS can be initiated in individuals aged 18–49 who have dyslipidemia or overweight/obesity conditions. However, for individuals over 50 years old, the most likely initiating component of MS was dyslipidemia or hypertension. People who initially had dyslipidemia were most likely to develop a combination of dyslipidemia with overweight/obesity before the age of 50, but after 50 years of age, people with dyslipidemia were most likely to have combined dyslipidemia and hypertension. Subjects (with the exception of males above 50 years of age who initially had an isolated state of hyperglycemia) who initially had an isolated state of overweight/obesity, hypertension, or hyperglycemia were most likely to develop a combination of one of these initial states with dyslipidemia. Males who initially had isolated hyperglycemia tended to develop hypertension after age 50. People who initially had isolated hyperglycemia or a 2-component state that contained hyperglycemia had a higher risk of developing MS than those who initially had other abnormal metabolic states.

## Abbreviations

MS: metabolic syndrome
CDS criteria: Chinese Diabetes Society criteria
CHD: coronary heart disease
BMI: body mass index
FPG: fasting plasma glucose
TG: triglyceride
HDL-C: high-density lipoprotein cholesterol
SBP/DBP: systolic/diastolic blood pressure
PG: 2 h post-meal glucose
FFA: free fatty acids
IR: insulin resistance
